# Hypercalcemia Induced by Tirzepatide and Calcium Supplementation: A Case Report

**DOI:** 10.7759/cureus.108061

**Published:** 2026-04-30

**Authors:** Edward Nguyen, Megan Kawamura, Irwin Munoz, Gary Ahn

**Affiliations:** 1 Internal Medicine, University of Hawaii Internal Medicine Residency Program, Honolulu, USA; 2 Internal Medicine, University of Hawaiʻi John A. Burns School of Medicine, Honolulu, USA; 3 Internal Medicine, Tripler Army Medical Center, Honolulu, USA; 4 Internal Medicine, The Queen's Medical Center, Honolulu, USA

**Keywords:** acute hypercalcemia, calcium supplement, dual incretin therapy, glp-1 agonist, tirzepatide

## Abstract

Tirzepatide is a glucagon-like peptide-1 receptor (GLP-1R) agonist and glucose-dependent insulinotropic polypeptide receptor (GIPR) agonist that is used for diabetes mellitus and weight loss. Common adverse effects include gastrointestinal symptoms such as nausea and vomiting, pancreatitis, and acute kidney injury. Hypercalcemia is rarely reported in the literature and has previously been reported in patients taking GLP-1R agonists with concomitant thiazide diuretic or exogenous calcium supplement. We report a 70-year-old female with obstructive sleep apnea started on tirzepatide three months prior to presentation, osteoporosis on calcium supplements, and obesity, who developed nausea and vomiting. She was found to have symptomatic moderate hypercalcemia with hypokalemia, metabolic alkalosis, and acute kidney injury that corrected after intravenous hydration. No prior literature describes hypercalcemia from tirzepatide use and calcium supplementation. The mechanism most likely involves tirzepatide’s effect on bone turnover and concomitant increased absorption from calcium supplement ingestion. Clinicians need to closely monitor patients’ electrolytes, especially calcium, when initiating patients on tirzepatide. Further research is warranted to reveal tirzepatide’s mechanism regarding calcium metabolism.

## Introduction

Tirzepatide is the first novel dual incretin glucagon-like peptide-1 receptor (GLP-1R) agonist and glucose-dependent insulinotropic polypeptide receptor (GIPR) agonist that has been approved to treat type 2 diabetes mellitus and obesity. It is currently the most widely prescribed GLP-1R agonist [1]. Incretins enhance insulin secretion post-meals, suppress glucagon release, delay gastric emptying, and reduce appetite [2,3]. Known adverse effects include prolonged satiety, appetite suppression, delayed gastric emptying, nausea, vomiting, diarrhea, gallstone disease, pancreatitis, retinal disorders, and thyroid-related disorders [3].

Hypercalcemia affects 1% of the worldwide population and is typically caused by elevated parathyroid levels or malignancy [4]. GLP-1R agonists and tirzepatide use have previously been associated with an increase in calcium levels. Alenezi et al. (2024) found that GLP-1R agonists are associated with an increased risk of hypercalcemia, and tirzepatide has an increased risk of 85% (RR: 1.85, 95% CI: 1.079-3.171) to develop hypercalcemia [5]. GLP-1R agonist use with other drugs that promote calcium levels in the blood has previously been shown to cause hypercalcemia. Nduma et al. (2025) showcased a patient who developed hypercalcemia while taking hydrochlorothiazide and tirzepatide [6]. Liraglutide and calcium supplements have also been suggested to cause hypercalcemia due to increased medication absorption due to slow gastrointestinal motility [7]. Here, we present a novel case of a patient taking calcium supplements for osteoporosis and started on tirzepatide to promote weight loss for obstructive sleep apnea (OSA), who later developed hypercalcemia, hypokalemia, and metabolic alkalosis.

## Case presentation

The patient is a 70-year-old female with a medical history of OSA, osteoporosis, obesity, hypertension, hyperlipidemia, and prediabetes who presented to the hospital for nausea and constipation. Three months prior to arrival, the patient was diagnosed with OSA and given three options for treatment: mandibular advancement device, continuous positive airway pressure (CPAP), or medical management with tirzepatide. The patient opted for medical management and was started on Tirzepatide 2.5 mg weekly. She initially had nausea for the first week, which resolved, and was transitioned to a higher dose of 5 mg the following month. Recurrence of nausea for the first week occurred, which eventually resolved. Her dose increased to 7.5 mg the following month. However, with this increased dose, her nausea continued to progressively worsen and did not resolve by the end of the first week. Despite this, she continued to take tirzepatide weekly, and her last dose was 1.5 weeks prior to her arrival at the emergency department. In the week prior to her presentation, she was unable to tolerate her usual food intake and ate half a sandwich at most with some milk. She also had episodes of dry heaving, but denies emesis with food content. She had occasional constipation, described as “hard stools,” but was still having daily bowel movements. She also endorsed increased urinary frequency, but attributed this to drinking more water. She lost approximately 20 pounds of weight and noticed improvements to her sleep, as she had been able to sleep throughout the night and woke up less frequently. Her other medications include amlodipine 5 mg daily, losartan 50 mg twice daily, atorvastatin 10 mg daily, aspirin 81 mg daily, calcium supplement 600 mg twice daily, multivitamin with 160 mg calcium daily, and vitamin D3 25 mcg daily.

**Figure 1:**
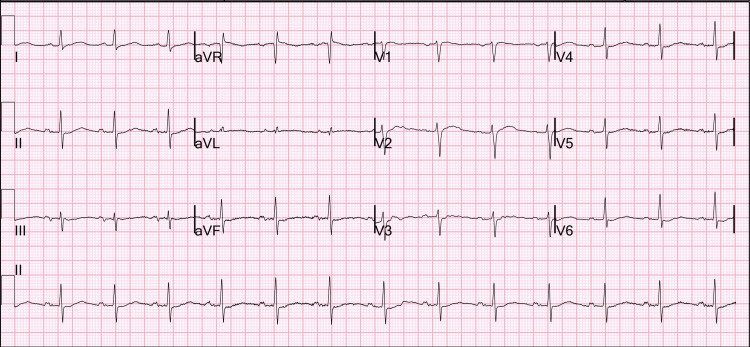
Electrocardiogram With Nonspecific T Wave Abnormalities

She contacted her primary care physician who obtained a basic metabolic panel, which was remarkable for hypokalemia at 2.9 mEq/L, elevated bicarbonate 38 mEq/L, elevated creatinine 1.1 mg/dL, and moderate hypercalcemia 13.1 mg/dL (Table 1). Her prior basic metabolic panel seven months ago was unremarkable with creatinine 0.6 mg/dL (Table 2). She was then encouraged to go to the emergency department, and her vitals were blood pressure 131/62 mmHg, pulse 72 beats per minute, temperature 36.2°C (97.2°F), respiratory rate 15 breaths per minute, and peripheral oxygen saturation (SpO_2_) 96% on room air. Her physical exam was grossly unremarkable. Electrocardiogram showed a normal sinus rhythm at 79 beats per minute and a nonspecific T wave abnormality (Figure 1). Laboratory studies were remarkable for severe hypokalemia at 2.2 mEq/L, elevated bicarbonate 39 mEq/L, elevated creatinine 1.0 mg/dL, moderate hypercalcemia 13.1 mg/dL, and hypophosphatemia at 1.5 mg/dL. Magnesium was normal at 1.8 mg/dL. Ionized calcium was elevated at 1.53 mmol/L. Thyroid-stimulating hormone (TSH) and parathyroid hormone (PTH) were normal at 1.14 uIU/mL and 15 pg/mL respectively. Total 25-hydroxy vitamin D was normal at 37.6 ng/mL. Parathyroid hormone-related protein (PTHrP) and 1,25-dihydroxy vitamin D were low at 8 pg/mL and 13 pg/mL, respectively (Table 3).

**Table 1:** Laboratory Values Obtained by Primary Care Physician Prior to Hospitalization

Parameters	Patient Values	Reference Range
Potassium	2.9 mEq/L	3.3-5.1 mEq/L
Bicarbonate	38 mEq/L	21-31 mEq/L
Creatinine	1.1 mg/dL	0.6-1.3 mg/dL
eGFR	54 mL/min/1.73m^2	>=90* mL/min/1.73m^2
Calcium	13.1 mg/dL	8.6-10.3 mg/dL

**Table 2:** Laboratory Values Obtained by Primary Care Physician Seven Months Prior to Hospitalization eGFR: estimated glomerular filtration rate.

Parameters	Patient Values	Reference Range
Potassium	4.1 mEq/L	3.3-5.1 mEq/L
Bicarbonate	30 mEq/L	21-31 mEq/L
Creatinine	0.6 mg/dL	0.6-1.3 mg/dL
eGFR	96 mL/min/1.73 m^2^	≥90 mL/min/1.73 m^2^
Calcium	9.7 mg/dL	8.6-10.3 mg/dL

**Table 3:** Initial Laboratory Values Obtained in the Hospital

Parameters	Patient Values	Reference Range
Potassium	2.2 mEq/L	3.3-5.1 mEq/L
Bicarbonate	39 mEq/L	21-31 mEq/L
Creatinine	1.0 mg/dL	0.6-1.3 mg/dL
eGFR	61 mL/min/1.73m^2	>=90* mL/min/1.73m^2
Calcium	13.1 mg/dL	8.6-10.3 mg/dL
Phosphorus	1.5mg/dL	2.6-4.9 mg/dL
Magnesium	1.8 mg/dL	1.5-2.5 mg/dL
Ionized Calcium	1.53 mmol/L	1.15 - 1.33 mmol/L
Thyroid Stimulating Hormone	1.14 uIU/mL	0.3-4.2 uIU/mL
Parathyroid Hormone	15 pg/mL	12-88 pg/mL
Total 25-Hydroxy Vitamin D	37.6 ng/mL	20-50 ng/mL
Parathyroid-Related Protein	8pg/mL	11-20 pg/mL
1,25-Dihydroxy Vitamin-D	13 pg/mL	18-72 pg/mL

In the emergency department, the patient was given 1 L lactated ringer bolus, 1 L normal saline bolus, 40 mEq potassium, 40 mg lasix, and 1 g magnesium sulfate. She then denied having any nausea or constipation. After she was admitted, she was started on maintenance intravenous fluids with lactated ringers at 300 mL with a goal urine output of 100 mL to 150 mL per hour. Her bicarbonate worsened to 41 mEq/L, and she also developed trace bilateral lower extremity edema (Table 4). Her fluids were subsequently changed to normal saline 100 mL/hr. Her potassium was trended and repleted as needed, and her calcium improved. Two days later, her electrolytes normalized (Table 4). She was discharged with instructions to stop taking the tirzepatide, calcium supplement, multivitamin, and vitamin D supplement.

**Table 4:** Laboratory Values During Hospital Course eGFR: estimated glomerular filtration rate.

Parameters	Day 1	Day 2
Potassium	3.0 mEq/L	3.4 mEq/L
Bicarbonate	41 mEq/L	34 mEq/L
Creatinine	0.9 mg/dL	0.8 mg/dL
eGFR	69 mL/min/1.73 m^2^	79 mL/min/1.73 m^2^
Calcium	11.5 mg/dL	9.5 mg/dL
Phosphorus	2.1 mg/dL	3.2 mg/dL
Magnesium	1.9 mg/dL	1.7 mg/dL
Ionized calcium	1.45 mmol/L	1.20 mmol/L

## Discussion

Hypercalcemia is categorized into different severities: mild, moderate, or severe. Mild hypercalcemia (serum calcium: <12 mg/dL or ionized calcium: 5.6-8.0 mg/dL) may be asymptomatic or present with constitutional symptoms such as fatigue and constipation [4]. Moderate hypercalcemia (serum calcium: 12.0-13.9 mg/dL or ionized calcium: >10 mg/dL) may present with generalized symptoms such as fatigue or anorexia; urinary symptoms such as polyuria or polydipsia; gastrointestinal symptoms such as nausea, vomiting, abdominal pain, or dyspepsia; musculoskeletal symptoms such as muscle weakness, arthralgias, myalgias, or bone pain; cardiac symptoms such as a shortened QT interval, prolonged PR interval, or bradydysrhythmias; or neuropsychiatric symptoms such as hyporeflexia, lethargy, altered mental status, anxiety, depression, and emotional instability [4,8]. Severe hypercalcemia (serum calcium: >14 mg/dL or ionized calcium: 10-12 mg/dL) may additionally present with confusion, stupor, or coma [4].

Before attributing the patient’s hypercalcemia to the combination of tirzepatide administration and calcium supplementation, more common causes of hypercalcemia were considered. Primary hyperparathyroidism was less likely because her parathyroid hormone (PTH) level was normal at 15 pg/mL. Humoral hypercalcemia of malignancy was less likely because her parathyroid hormone-related protein level was normal at 8 pg/mL. Hypervitaminosis D was less likely because her 25-hydroxy vitamin D (25-OH Vit D) was normal at 37.6 ng/mL and her 1,25-dihydroxy vitamin D was low at 13 pg/mL (reference: 18-72 pg/mL). Multiple myeloma was less likely, as she did not have renal failure, anemia, or bone pain. Hyperthyroidism was less likely, as her TSH was normal at 1.14 uIU/mL. Thiazide diuretics may also cause hypercalcemia, but this patient was not on these medications.

Given the limited studies on tirzepatide-induced hypercalcemia and known calcium supplementation in this patient, this combination was the most likely culprit that caused this patient’s symptomatic moderate hypercalcemia. Her symptoms began after three months on escalating doses of tirzepatide therapy, which is consistent with what Alenezi et al. reported in their study [5]. Furthermore, the patient’s concomitant daily calcium supplementation (600 mg twice daily + 160 mg in daily multivitamin) with milk intake may have induced mild, asymptomatic hypercalcemia that persisted for several weeks before presenting with constipation, urinary frequency, nausea, vomiting, and decreased appetite.

The mechanism of GLP-1R agonists and GIPR agonists' effects on the development of hypercalcemia is unclear. Calcium homeostasis is regulated by the complex interplay between hormones such as PTH and calcitonin, vitamin D, the calcium-sensing receptor (CaSR), and their effects on the bones, intestines, and kidneys. There is a paucity of literature looking at the impacts of tirzepatide or other GLP-1R agonists on PTH, vitamin D levels, or CaSR activity [9]. Several studies have endorsed both GLP-1R agonists’ and GIPR agonists’ roles in promoting bone formation through promotion of osteoblast activity while inhibiting osteoclastic activity, which would theoretically lead to hypocalcemia, contrary to what was observed with this patient [10,11]. This suggests that there may be alternative mechanisms besides GLP-1R and GIPR agonists’ effects on bone metabolism and calcium homeostasis in this patient.

Previous literature has shown that use of GLP-1R agonists and thiazide diuretics or calcium supplementation can contribute to hypercalcemia [5,6]. Although this patient was not on a thiazide diuretic, increased calcium absorption from her calcium supplement could have contributed to her hypercalcemia. This patient’s clinical presentation suggests that she may have had milk-alkali syndrome, given her hypercalcemia, metabolic alkalosis, and elevated creatinine. However, she did not meet the 4-5 g ingestion of calcium carbonate daily that is typically associated with milk-alkali syndrome [12]. In order for hypercalcemia to develop in milk-alkali syndrome, the patient may either ingest "excessive" amounts of calcium or have an impaired ability to excrete calcium. Tirzepatide’s slowed gastric emptying effects may have allowed for increased absorption of her calcium supplements. Perhaps it may also impair calcium carbonate excretion, which led to the patient’s hypercalcemia and alkalosis. Further research should be done to elucidate the mechanism behind GLP-1R and GIPR agonist-induced hypercalcemia.

The link between hypercalcemia, hypokalemia, and metabolic alkalosis has been well-studied. Hypercalcemia can induce hypokalemia through inhibition of sodium-potassium-chloride (NKCC2) channels in the thick ascending limb of the kidney, which results in increased sodium and potassium excretion with contraction alkalosis [13]. Consequently, there is increased delivery of sodium-rich filtrate to the collecting duct, which results in enhanced sodium reabsorption with secretion of H+ and potassium [13,14]. The resultant alkalosis and hypokalemia becomes a self-perpetuating cycle. Depletion of potassium stores causes intracellular acidosis, leading to increased H+ secretion in the proximal convoluted tubule (PCT), distal convoluted tubules (DCT), and collecting duct. There is also an associated increase in bicarbonate reabsorption and stimulation of ammonia production in the PCT. In the DCT and collecting duct, H-K ATPases and H-ATPases increase in activity, which rid of H+ while reabsorbing more bicarbonate [15]. Taken together, this suggests that this patient’s hypercalcemia from concomitant tirzepatide and calcium supplementation resulted in initial symptomatic hypercalcemia with consequent hypokalemia and metabolic alkalosis.

## Conclusions

This case highlights the importance of monitoring electrolytes at baseline and in subsequent weeks in patients starting on tirzepatide, especially in those who may also be taking calcium supplements or medications including thiazide diuretics. The patient’s initial symptoms of nausea and vomiting during dose escalation are common side effects of GLP-1R agonist therapy and made it difficult to recognize the patient's hypercalcemia until it persisted beyond the one-week course on the 7.5 mg dose. Thus, providers should exercise caution when prescribing GLP-1R agonists such as tirzepatide, even in patients with no prior history of renal disease, malabsorptive disorders, or hypercalcemia-inducing medications, because electrolyte abnormalities may still occur. Additional research is needed to uncover the mechanism of hypercalcemia and tirzepatide use.
